# Vegetarian Diet: An Overview through the Perspective of Quality of Life Domains

**DOI:** 10.3390/ijerph18084067

**Published:** 2021-04-12

**Authors:** Shila Minari Hargreaves, António Raposo, Ariana Saraiva, Renata Puppin Zandonadi

**Affiliations:** 1 Department of Nutrition, Faculty of Health Sciences, University of Brasilia (UnB), Campus Darcy Ribeiro, Asa Norte, Brasilia, DF 70910-900, Brazil; renatapz@unb.br; 2 CBIOS (Research Center for Biosciences and Health Technologies), Universidade Lusófona de Humanidades e Tecnologias, Campo Grande 376, 1749-024 Lisboa, Portugal; 3 Department of Animal Pathology and Production, Bromatology and Food Technology, Faculty of Veterinary, Universidad de Las Palmas de Gran Canaria, Trasmontaña s/n, 35413 Arucas, Spain; ariana_23@outlook.pt

**Keywords:** quality of life, quality of life domains, vegetarian diet, vegetarianism

## Abstract

Vegetarianism has gained more visibility in recent years. Despite the well-described effects of a vegetarian diet on health, its influence on the quality of life of the individuals who follow it still needs to be properly investigated. Quality of life relates to a subjective perception of well-being and functionality, and encompasses four main life domains: physical, psychological, social, and environmental. The adoption of a vegetarian diet, despite being a dietary pattern, could potentially influence and be influenced by all of these domains, either positively or negatively. This review aims to present an overview of the background, conceptualization, features, and potential effects of vegetarianism in all quality of life domains. The choice of adopting a vegetarian diet could have positive outcomes, such as better physical health, positive feelings related to the adoption of a morally correct attitude, an increased sense of belonging (to a vegetarian community), and lower environmental impact. Other factors, however, could have a negative impact on the quality of life of those choosing to abstain from meats or other animal products, especially when they go beyond one’s control. These include the environment, the social/cultural group in which a person is inserted, gender-based differences, economic aspects, and a limited access to a wide variety of plant-based foods. It is important to understand all the effects of adopting a vegetarian diet—beyond its nutritional aspects. Not only do studies in this area provide more consistent data, but they may also contribute to mitigating all factors that might prevent individuals from adopting a vegetarian diet, or that may have a negative impact on the quality of life of those who already follow it.

## 1. Introduction

Vegetarianism has its origins in 3200 BC, when ancient Egyptian civilizations started adopting vegetarian diets based on the belief that abstaining from meat consumption would facilitate reincarnation [1]. In India, another important cradle of vegetarianism, this practice was also associated with the fact that Hindus see cows as sacred and uphold nonviolence principles [2]. Later, Greek philosophers also adopted a vegetarian diet, with Pythagoras being a leading figure among them—indeed, for many centuries, vegetarianism was known as the “Pythagorean” diet [3,4]. In the Christian Era, vegetarianism lost its strength, gaining some visibility again only in the late 18th and early 19th centuries, when Darwin’s theory of evolution challenged the Church’s views that animals had no souls, and that their only purpose on Earth was to serve human beings [1,5].

Throughout history, the expansion of vegetarianism has been associated with religions that preach respect for all living beings and adopt nonviolence principles, such as Hinduism, Jainism, Sikhism, Buddhism, the Hare Krishna movement, and the Seventh-day Adventist Church. In addition, in the 20th and 21st centuries, science has observed several health benefits potentially associated with the reduction in meat consumption. Such benefits have strengthened the practice of vegetarianism around the world, and attracted more and more followers [4].

Currently, the worldwide prevalence of vegetarianism is not uniform. Asia is the continent with the highest prevalence, with 19 percent of the population adopting this practice [6]. India, the single country with the highest prevalence in the world (almost 40 percent of the population), contributes to the results of the Asian continent [7]. The prevalence in Africa and the Middle East is about 16 percent; and in Central and South America, 8 percent. The lowest prevalence of vegetarianism is found in North America (about 6 percent of the population are vegetarians) and Europe, where vegetarianism is adopted by only 5 percent of the population. 

Vegetarianism encompasses different types of diets, classified according to how restrictive they are. Generally, vegetarianism is understood as the exclusion of meat from one’s diet, but other less restrictive eating patterns can also be classified within the scope of vegetarianism. These include, for example, flexitarians, who consume meat sporadically, or even once a week; pescatarians, who avoid all meat, except fish and seafood; and ovolactovegetarians, who banish all types of meat but consume products of animal origin, such as eggs and dairy products. A strict vegetarian diet, on the other hand, excludes all foods of animal origin. Veganism is a broader concept, which involves the adoption of a strict vegetarian diet, as well as the exclusion of other consumer items made from animal products, or which rely on animal exploitation, such as cosmetics and clothing items [8,9]. For didactic purposes, a strict vegetarian diet is often referred to as a vegan diet.

Different motivations can lead to adopting a vegetarian diet [10,11,12,13]. Ethical concerns are the main reasons, building on the idea that animal slaughter for human consumption is morally inappropriate. Another important motivation is health and the potential beneficial effects of vegetarianism. Religions that encourage abstaining from meat consumption and concerns about the environmental impacts of meat production are also important motivators for adopting vegetarianism [7,9].

According to the World Health Organization (WHO), quality of life (QoL) is a subjective concept that comprehends physical, psychological, social, environmental, and spiritual aspects [14,15]. Changes in eating patterns can influence individuals’ QoL, both positively and negatively [16]. A systematic review study assessed the nutritional quality of vegetarian diets, and found—based on data from 12 surveys—higher nutritional quality levels among vegetarians than omnivores [17]. According to the Academy of Nutrition and Dietetics [18], vegetarian diets are nutritionally adequate for all stages of life, as long as they are well planned. However, some precautions need to be taken to minimize the risk of nutritional deficiencies.

In view of the recent growth in the number of individuals adopting a vegetarian diet, as well as the wider interest in the topic in recent years, it is critical to understand the different effects of vegetarianism on one’s QoL. Therefore, this review aims to present an overview of the background, conceptualization, features and potential effects of vegetarianism considering all QoL domains.

## 2. Historical Background of Vegetarianism

Over most of their 24 million years of evolution, humans’ anthropoid ancestors were almost exclusively vegetarian, except for the occasional ingestion of insects and larvae. Anatomically, both humans and their ancestors present significant features that distance them from meat-eating animals, including, for example, wide flat teeth and more mobile jaws, which facilitate the chewing of grains and seeds, as opposed to sharp teeth and jaw movements on a vertical axis, which are characteristic of carnivores. In addition, carnivorous animals have shorter intestines, which enable the rapid elimination of toxins, unlike humans and other predominantly herbivorous animals, with long intestines that allow longer digestion, fermentation and absorption processes [19,20].

However, possibly due to other reasons linked to survival, self-defense and territorial protection, hominids began hunting other species, which led to the introduction of meat in the diet of *Homo erectus*, considered the first hunters. Humans’ ability to survive on different types of food was an essential factor in our evolution, which allowed our species, *Homo sapiens sapiens*, to adapt to the most diverse conditions and spread throughout the planet [19,20].

During the Paleolithic era, different food types were consumed, such as wild plants, seafood, reptiles, birds, and mammals. After the emergence of agricultural practices (about 13,000 years ago), there is no evidence that humans were essentially vegetarian, and the domestication of animals, including for consumption, became a routine activity by that time. However, it is speculated that many farmers lived primarily as vegetarians due to the wider availability of crops [19].

It is not known for certain when people started voluntarily abstaining from meat. However, the first reports date from 3200 BC in ancient Egypt, when the practice was motivated by religious factors, based on the belief that not consuming meat would facilitate reincarnation [1]. Another important region that is part of the history of vegetarianism is India, where the practice is also linked to religious issues. Hinduism has two basic principles among its foundations: ahimsa, or the principle of nonviolence (which includes violence against humans and other animals); and the recognition of the cow as a sacred animal [2].

Some of the philosophers of the pre-Christian era also contributed to the spread of vegetarianism. The practice was adopted at that time for health reasons as well as for religious, ecological, and philosophical reasons. It was believed that the act of killing another living being for food would have a brutal influence on one’s mind, negatively affecting one’s body and soul [3]. The supporters of vegetarianism included big names like Plato, Prophyry, Diogenes and Plutarch. The most prominent philosopher in this field was Pythagoras, who lived in the 6th century BC. Due to his influence, vegetarianism was known as the “Pythagorean” diet over many centuries, a name that lasted until the middle of the 19th century in Europe and the Mediterranean region [4,19].

In Ancient Greece, it was believed that animals could think and communicate, and that humans should be responsible for their lives. In addition, the Greeks believed that eating meat would be harmful to one’s health and mind [21]. Vegetarianism was also present during the Roman Empire, influenced by the Greek culture. However, with the rise of Christianity, abstaining from animal consumption lost its importance. Famous Christian thinkers such as Saint Thomas Aquinas and Saint Augustine sought to provide rational justifications for the exploitation and consumption of animals, spreading the idea that, unlike animals, human beings have souls and free will, and that animals are inferior beings, placed on Earth at the service of humans [3,4,5]. Only a few monks still maintained the practice, based on the belief that meat consumption would hinder their spiritual progress in some way because it was linked to impulsive behaviors [5].

In the 15th century, vegetarianism was advocated by Leonardo da Vinci, who believed that there was no distinction between the murder of humans and animals. However, it was only after the spread of Darwin’s theory of evolution that vegetarianism gained strength again in the late 18th century and early 19th century. Darwinism refuted the idea that human beings are fundamentally different from other animals—therefore, there were no plausible justifications for meat consumption [5]. At that time, the first vegetarian societies also began to emerge, and some Christian groups began to preach in favor of abstaining from meat based on the belief that animals should also be worthy of pity. It was only then that the term “vegetarianism” came to be used. Despite the general belief that it refers to “eating vegetables”, the term actually derives from “vegetus”, a Latin word that means “active” or “vigorous” [22]. An important name in the history of vegetarianism, in addition to the various vegetarian groups and societies that emerged in the 20th century, was Mahatma Gandhi, who contributed to its dissemination [19].

Albert Einstein believed that humanity’s evolution toward a vegetarian diet would be fundamental for the survival of life on Earth [21]. In Europe, the first International Vegetarian Union was founded in 1908, after other vegetarian societies had already emerged in several countries. From the 1960s onwards, a greater concern with food and health, associated with evidence of the potential benefits of a vegetarian diet for disease prevention, contributed to the spread of vegetarianism. Religious practices that preach respect for life and adopt nonviolence principles, such as Hinduism, Jainism, Sikhism, Buddhism, the Hare Krishna movement, and the Seventh-day Adventist Church, were also fundamental to this growth. Therefore, the world has seen a significant rise and expansion of the practice since the mid-20th century [4].

In recent years, vegetarianism has gained more visibility and a greater number of followers. Rosenfeld [23] describes a great expansion in the scientific literature on the psychological and social effects of choosing a vegetarian diet. Some topics started to attract more attention, such as motivations; barriers to adopting such diets; differences between vegetarians and vegans; morality; and gender differences. New research lines have emerged to explore issues associated with personal identity and social and cultural experiences [23].

Adherence to a vegetarian diet goes beyond food. Vegetarianism can be considered a social identity, as it reflects the motivations, feelings, and attitudes of those who choose to adopt it [24]. The main motivations for choosing a vegetarian diet are related to ethical and health aspects. Animal welfare is the main motivator, followed by concerns with major environmental impacts caused by the production and consumption of food of animal origin. Regarding health, general well-being and weight maintenance are the factors that most motivate the adoption of vegetarianism [23]. In addition, religious aspects can lead individuals to adopt a vegetarian diet, and religions such as Hinduism, Adventism and Spiritism preach abstaining from meat. Other less frequent factors, such as aversion to the taste of meat, food intolerances and allergies, and the influence of other people (family members, for example) can also be considered motivators for adopting a vegetarian diet [4,7,9,21].

There are several types of vegetarian diets commonly described in the literature. The most consensual classification consists of four different types, namely: (1) flexitarian or semivegetarian diet, in which people consume meat sporadically (up to once a week) or exclude red meat, but consume white meat; (2) pesco-vegetarian or pescatarian diet, which excludes all meats, except fish and seafood; (3) ovolactovegetarian diet, which excludes all types of meat, but allows products of animal origin, such as dairy products and eggs; and (4) strict vegetarianism, which excludes all products of animal origin [8,25].

In addition to these categories, other diets can be considered subclassifications of vegetarianism, namely: (1) raw vegan diet, which is mostly based on food in its most natural (raw) state, with an emphasis on the choice of organic and self-grown products; (2) frugal or frugivorous diet, which is similar to the raw vegan diet, but with 70–80 percent of the diet being composed of fruits, with a small proportion of nuts, seeds and some vegetables; and (3) macrobiotic diets, which encompass various degrees of restriction but are primarily composed of whole grains, soybeans, algae and some vegetables [25,26].

## 3. Quality of Life

According to the WHO, QoL is a multifactorial concept that includes the following domains: physical (physical state), psychological (affective and cognitive state), social (interpersonal relationships and social roles in the lives of individuals) and environmental (quality of the environment in which individuals live). Conceptual, pragmatic and empirical dimensions, as well as spiritual and religious aspects, can also contribute to people’s QoL and their ability to perform certain activities, or “functionality”. Building on that, QoL is defined as “individuals’ perception of their position in life in the context of the culture and value systems in which they live and in relation to their goals, expectations, standards and concerns” [14,15].

The terms “quality of life” and “well-being” are often used to indicate how well an individual feels. There is, however, a problem of interpretation resulting from the subjectivity of these concepts, which may acquire a broader or more specific connotation depending on the context. QoL can be subdivided into: the quality of the environment in which one lives, involving the physical structure of the environment and people’s integration in the society in which they live; physical and mental health, encompassing a wide range of individual capacities; usefulness, which involves the feeling of “being useful”, contributing to the welfare of other people, society, and the environment; and the appreciation of life, which is associated with tangible (wealth, for example) and intangible (such as life satisfaction and happiness) aspects [27].

Although it is difficult to group all these qualities into a single concept, the best general indicator of QoL would be how happy you feel and how long you live. The concept of “well-being”, in turn, usually denotes QoL in a wider sense, as well as a positive subjective assessment of life, or an appreciation of life. However, sometimes the concepts of “well-being” and “quality of life” are used interchangeably [27].

**Figure 1:**
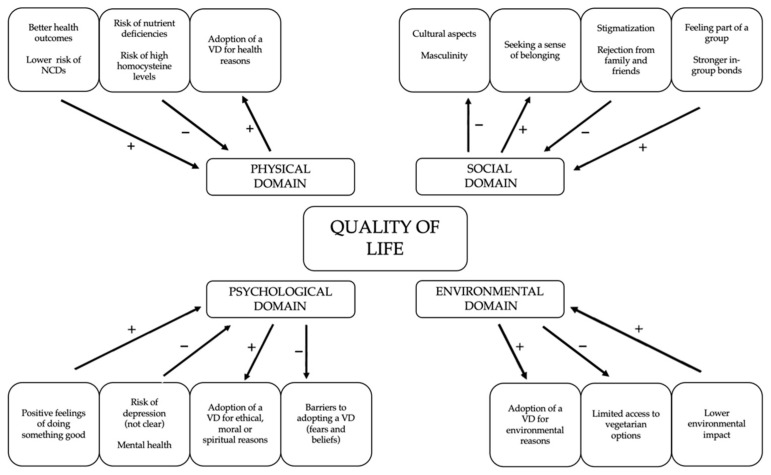
Connections between aspects of vegetarianism and quality of life domains. The arrows indicate the direction of the influence, that is, whether a given domain influences or is influenced by certain aspects of vegetarianism. The plus (+) and minus (−) symbols indicate positive and negative influences, respectively. NCD: noncommunicable diseases; VD: vegetarian diet.

The connection between vegetarianism and QoL may be analyzed through different perspectives [14,15]. In the context of vegetarianism, each QoL domain proposed by the WHO (physical, psychological, social, and environmental) may be influenced by the adoption of a vegetarian diet. The opposite may also be said, that is, specific aspects of each domain might influence one’s decision to adopt a vegetarian diet. Moreover, these influences could be either positive or negative. The possible connections between vegetarianism and QoL domains are illustrated in Figure 1.

### 3.1. Physical Domain

The physical domain refers to aspects as pain, discomfort, energy, fatigue, sleep, and rest. Aspects that positively contribute to a general feeling of physical well-being are therefore relevant for understanding QoL. These include better general health, lower rates of chronic and inflammatory diseases, and lifespan [28].

#### 3.1.1. Influence of Adopting a Vegetarian Diet on the Physical Domain

##### Positive Influence

Following a vegetarian diet may lead to better health outcomes and a lower risk of noncommunicable diseases, which could positively influence the QoL physical domain (Figure 1). A nutritionally adequate diet is essential to achieving and maintaining good overall health. A systematic review published by Parker and Vadiveloo [17] compared the quality of vegetarian and nonvegetarian diets based on diet quality indexes. That review included 12 studies and showed that vegetarians have better diet quality results than omnivores. Furthermore, among vegetarians, vegans achieved the best results. Although different indexes were used in the studies, several common points allowed a combined analysis of the results. Higher consumption of fruits, green vegetables, whole grains, and vegetable sources of protein—and lower consumption of saturated fat and sodium—contributed to the best results found among vegetarians [17].

A cross-sectional study carried out with vegetarians in Brazil (n = 3319) observed that vegetarians have better diet quality markers than the general Brazilian population, according to parameters used in a national annual survey carried out by the Ministry of Health [29,30]. It was observed that a higher proportion of vegetarians had a more adequate daily consumption of fruits and vegetables [29] compared to the general Brazilian population (38.1 percent versus 23.1 percent), based on WHO recommendations (five servings a day) [31]. In addition, a lower regular weekly consumption of soft drinks and artificial juices was also observed among vegetarians (3.9 percent versus 14.4 percent). Of the different types of vegetarians, vegans showed the best results. It was also observed that vegetarians in Brazil follow the recommendations set out in the Dietary Guidelines for the Brazilian Population with regard to consuming more fresh foods and fewer processed and ultraprocessed foods [32]. 

Vegetarian diets, including strict vegetarianism (veganism), are considered healthy and nutritionally adequate, and can supply people’s nutritional needs at all life stages, as long as such diets are well planned [18]. Moreover, the benefits related to the prevention and better control of chronic diseases among vegetarians have already been described, and could also lead to positive outcomes in their QoL.

The role of intestinal microbiota in the regulation of several biological functions and in the prevention of chronic diseases is well known, as well as the fundamental role of the diet in the microbiota and intestinal health of individuals [33,34,35]. Excessive protein consumption could alter intestinal microbiota patterns by stimulating the proliferation of bacteria capable of fermenting amino acids. Such fermentation results in the production of molecules responsible for increased intestinal permeability, inflammation, and even cancer [36]. The consumption of vegetable sources of protein, on the other hand, is not associated with such adverse effects, possibly because they contain carbohydrates and fibers, which could mitigate the potentially deleterious effects observed in the intestine caused by the ingestion of proteins [36]. The intake of saturated fats, present mainly in animal foods, is another factor that contributes to an increase in systemic inflammation, possibly through the activation of Toll-like receptors (TLR), which, once activated, trigger a proinflammatory intestinal and systemic immune response [37]. The activation of TLRs and the subsequent inflammatory cascade result in an increased risk of metabolic disorders and chronic diseases, such as cancer, insulin resistance, and cardiovascular diseases [37].

Vegetarian diets usually have a higher content of carbohydrates and fibers, in addition to lower levels of proteins and fats—in particular saturated fats. Studies comparing the microbiota of vegetarians and nonvegetarians show that a plant-based diet can benefit the diversity and profile of the bacteria that make up the intestinal microbiota. In addition to differences observed in the microbiota, with a more favorable bacterial profile, a vegetarian diet (with high consumption of whole foods, fruits, and vegetables) leads to increased production of metabolites from the fermentation of prebiotics and phytochemicals by these bacteria, which also have a positive effect on the host’s health, both at intestinal and systemic levels, contributing to the prevention of chronic diseases [38].

Among chronic diseases, cardiovascular diseases account for 43.6 percent of deaths worldwide [39]. Positive results in the control of cardiovascular disease risk factors were observed in clinical trials that promoted lifestyle changes, including adopting vegetarian, vegan, and plant-based diets [40,41,42,43]. A review of observational studies conducted in 2018 assessed cardiovascular risk factors in vegans. In most countries, vegetarian diets were associated with a lower intake of energy and saturated fat, and a better cardiovascular profile (lower body weight, LDL cholesterol levels, blood pressure, fasting glucose, and triglycerides) [44]. 

A 2019 review study conducted by the Diabetes and Nutrition Study Group (DNSG) of the European Association for the Study of Diabetes (EASD) associated vegetarian eating patterns with a 28 percent reduction in the incidence of coronary heart disease, and a 22 percent drop in mortality from such conditions. That study gathered data from systematic reviews with meta-analyses correlating different dietary patterns and cardiometabolic outcomes in diabetic patients [45]. Following a balanced vegetarian diet can reduce systemic inflammation and the risk of diabetes, two factors that are closely linked to the onset and progression of cardiovascular disease [46].

The consumption of refined carbohydrates, saturated fats, processed meats, and sugary drinks increases the risk of type-2 diabetes, especially when combined with low consumption of dietary fibers. On the other hand, a low-calorie plant-based diet has a protective effect [47]. 

The prevalence of diabetes among vegetarians is 1.6 to 2 times lower than among omnivores [48]. In a 24-week controlled trial with diabetics, the individuals who followed a vegetarian diet showed greater weight loss (6.2 kg versus 3.2 kg, on average), better insulin sensitivity (30 percent versus 20 percent), greater reduction in visceral fat and medication use, in addition to a better hormonal profile (increased adiponectin and reduced leptin) and better levels of antioxidants, as compared to the ones following a standard diet for diabetes control [49].

Several factors contribute to the reduction in risks and a better control of diabetes. The first one is vegetarians’ better weight control. It is known that both obesity and the accumulation of visceral fat are linked to increased insulin resistance, which contributes to the onset of diabetes [47]. Vegetarians’ lower intake of saturated fats [17] also contributes to reducing the risk of diabetes. It has been shown that reducing the consumption of saturated fats or replacing them with unsaturated fats may contribute to improving insulin sensitivity [50]. Other factors, such as higher fiber intake [51], lower ferritin levels and lower intake of heme iron [52] among vegetarians are also related to better insulin resistance and lower risk of diabetes.

A vegetarian diet may also contribute to improving inflammation control. Foods of plant origin—when consumed in their most natural form—are rich in antioxidants, which can assist directly in the control of free radicals in the body (as in the case of antioxidant vitamins C and E), or even through several signaling pathways that modulate our immune response and the production of antioxidant compounds and enzymes, suppressing inflammatory responses [48,53,54]. Therefore, a plant-based diet that is rich in fruits, vegetables, whole grains, seeds, and nuts can help to control inflammatory processes.

A vegetarian diet may also bring benefits regarding cancer prevention. In addition to vegetarians’ better weight control results [55], which can be considered a protective factor against cancer [56], their higher consumption of dietary fibers could have protective effects due to the modulation of the intestinal microbiota. In addition, as previously described, excessive protein consumption can lead to an increased production of inflammatory metabolites by the intestinal microbiota [36], and the consumption of saturated fats (found mainly in foods of animal origin) is capable of activating Toll-like receptors in immune system cells. This stimulates the production of proinflammatory cytokines [37], and all these factors together can create a cancer-promoting environment. 

In addition to the most common chronic diseases mentioned above, adopting a vegetarian diet can help to prevent and treat other inflammatory diseases. A healthier microbiota, higher consumption of antioxidants and lower consumption of potentially inflammatory compounds, in addition to better weight control, are important factors that positively contribute to the health of vegetarians. In fact, how long an individual has been following a vegetarian diet may have an important influence on their results—which depend on continuous exposure to this type of dietary pattern. In a study that evaluated only individuals who had been on a vegetarian diet for at least 15 years (n = 45), lower levels of oxidative stress markers were observed compared to omnivorous individuals (n = 30) [57].

Furthermore, promising results have already been achieved with the adoption of a vegetarian diet by individuals suffering from fibromyalgia, for example, including improvements in pain symptoms, QoL, sleep quality, and anxiety depression [58]. In autoimmune diseases, such as rheumatoid arthritis, a diet rich in fruits, vegetables, whole grains and legumes—and low in animal foods—can help to control some of the symptoms [59]. A vegetarian diet could also be a beneficial tool to prevent other autoimmune diseases, such as multiple sclerosis [60], due to its role in the health of the intestinal microbiota [61].

Several factors related to lifestyle may influence the emergence of diseases and how long an individual can live. Habits such as regular physical activities, stress control, good personal relationships, and a balanced diet have a positive impact on longevity [62]. A more detailed analysis of the dietary patterns followed by the world’s longest-living populations, who live in regions known as Blue Zones, can help us understand important food-related aspects that might contribute to improving people’s health and life expectancy. The five regions considered Blue Zones are: Loma Linda (California—United States), Nicoya (Costa Rica), Sardinia (Italy) Ikaria (Greece), and Okinawa (Japan). In all of them, individuals adopt a predominantly plant-based diet, with sporadic meat consumption (on average five times a month, in small portions). On the other hand, the consumption of legumes is frequent in all of them, being part of their daily diet, in addition to vegetables, tubers, cereals, fruits, and other regional foods, including dairy products [63]. 

The increased consumption of fruits and vegetables—rich in phytochemicals—may contribute to longevity through several mechanisms. The control of low-grade inflammation provided by antioxidant protection can prevent cell structure damage, slowing down the aging process [64]. On the other hand, prioritizing the consumption of proteins from animal sources could have a negative impact on one’s life expectancy. The profile of the amino acids found in these foods, with a higher content of methionine and branched-chain amino acids, leads to greater stimulation of IGF-1 and mTOR, in addition to greater cell proliferation. This contributes to the cellular senescence process and, consequently, to aging [65,66,67,68].

These potential health benefits of consuming a mostly or strictly plant-based diet can contribute to better physical health and well-being, resulting in better QoL. In fact, a cross-sectional study conducted with a total of 4628 individuals in the United Kingdom (with a wide range of diseases and conditions) showed that people who were ill had lower QoL scores than those feeling well. Post hoc comparisons indicated higher differences in the physical domain, especially among patients with musculoskeletal conditions ﻿(arthritis/arthroplasty, chronic pain), and those with cardiovascular disease awaiting a heart transplant [69]. Therefore, a diet that helps to prevent chronic and inflammatory diseases could also reduce the negative effects of these conditions on people’s QoL.

##### Negative Influence

Despite the potential health benefits from adopting a vegetarian diet, special attention should be given to the adequacy of iron, zinc, vitamins B12 and D, calcium, iodine, omega-3, and protein in adults [70], and especially in infants [71]. Low intake of such nutrients could lead to nutritional deficiencies and impair an individual’s health [70,72], with a negative impact on their QoL. 

Vitamin B12 deficiency should be highlighted, as this nutrient can only be found in animal-origin foods. Vegetarians (especially vegans) have been shown to have lower levels of serum vitamin B12. In addition, increased homocysteine levels [73,74,75] are observed, a metabolite that is elevated due to deficiency of vitamin B12 (and other nutrients), and which is associated with increased inflammation. B12 deficiency and increased homocysteine can lead to neurological problems, anemia and developmental delay in children, in addition to increasing the risks of cardiovascular disease, dementia, osteoporosis and death [73,75]. For this reason, it is necessary to monitor and supplement vitamin B12 levels among this groups, and possibly encourage the intake of fortified foods.

Iron, an essential mineral used for hemoglobin formation and oxygen transport in the body, also needs to be carefully adjusted. Vegetarians have been shown to have lower serum ferritin levels, a protein responsible for storing iron in the body. Lower levels of iron could increase the risk of developing anemia [76], which might also be caused by vitamin B12 deficiency [75]. In this scenario, an inadequately planned vegetarian diet could negatively affect aspects related to “energy and fatigue” in the physical domain of QoL [28].

Bone health should also be addressed when considering the potential negative effects of a vegetarian diet. A systematic review published in 2019 showed that vegetarians and vegans had lower bone mineral density than omnivores, and vegans also had higher fracture rates. Such results were unlikely explained only by lower calcium intake, as bone health encompasses many complex mechanisms and depends on different nutrients [77]. A recent cross-sectional study also found lower bone health in vegans when compared to omnivores (measured using quantitative ultrasound—QUS) [78], which reinforces the need for proper diet planning and careful bone health monitoring among vegetarians. 

#### 3.1.2. Influence of the Physical Domain on the Adoption of a Vegetarian Diet

##### Positive Influence

Seeking health improvement is one of the reasons why people chose to adopt a vegetarian diet [7]. According to Hopwood et al. [79], health was the most common reason why nonvegetarians considered adhering to a vegetarian diet. Vegetarianism is currently being more widely studied, and a growing number of scientific papers about the topic have been published over the past few years [80]. Consequently, the topic has received more attention from the media, and more information is reaching the general population. As more people are informed about the health benefits of adopting a vegetarian diet, the need or desire to improve their health might serve as a trigger. A study conducted in Germany with 329 vegans showed that more than two-thirds of them (69.6 percent), despite having more than one motive for following the diet, included health and well-being among them [81].

In this sense, following a vegetarian diet is both the cause and consequence of the positive outcomes related to the physical domain. People who seek health improvement may be prone to adopting a vegetarian diet; and, once they do it, the physical benefits may serve as further motivation for maintaining their new diet.

### 3.2. Psychological Domain 

The psychological domain is related to positive or negative feelings, self-esteem and body image/appearance, and thinking/learning/memory/concentration. Different aspects of vegetarianism can either influence or be influenced by psychological factors (Figure 1) [28].

#### 3.2.1. Influence of Adopting a Vegetarian Diet on the Psychological Domain

##### Positive Influence

Avoiding meat and other animal products can enhance positive feelings arising from the fact that person is adopting an attitude that confirms their beliefs. The positive psychological impact goes beyond the individual sphere, as it can also increase social connections with others adopting similar ideas and behaviors. According to Rosenfeld and Burrow [24], being a vegetarian goes beyond the choice of a dietary pattern, as it gives individuals a new social identity, which influences their way of thinking, behaving, and socializing. The adoption of a plant-based diet can have a positive effect on well-being and contentment, which could positively impact someone’s QoL [82].

The different motivations for adopting vegetarianism are also able to influence individuals psychologically. Those who adopt vegetarianism for ethical reasons tend to create more aversion to meat due to the association between its consumption and animal suffering. Such individuals also exclude more animal foods and tend to adopt stricter diets than those who become vegetarians for health or environmental reasons [23]. That does not necessarily implicate a negative outcome, though. As it has been shown by Cruwys et al. [83], vegetarians and vegans are more likely to report no barriers to diet adherence (25.2 percent of vegans and 15.6 percent of vegetarians) when compared to individuals following a gluten-free, paleo, or weight-loss diet. Indeed, both vegans and vegetarians had higher diet adherence when compared to the other groups, which might be connected to positive psychological effects related to the social identification within the vegetarian/vegan community.

##### Negative Influence

Potentially negative outcomes of vegetarianism in the psychological domain could be related to mental health impairment. The data related to the effect of vegetarianism on mental health are conflicting. Adopting a vegetarian diet was positively associated with a better mood in a cross-sectional study with Seventh-day Adventists [84]. A study of South Asians living in the United States found that the likelihood of depression was 43 percent lower among vegetarians [85]. However, a contrary association has also been observed: in the United Kingdom, a positive association of depressive symptoms was found in men, even after adjusting for confounding factors such as nutritional deficiencies and sociodemographic data [86]. Similar results were found among adolescents in a study conducted in Turkey, in which higher levels of anxiety, as well as eating disorders, were observed. That study raises the possibility that a vegetarian diet might be adopted among young people as a way of limiting food intake, and that it might be related to preexisting eating disorders [87].

Discrepant results have already been observed in a study that evaluated mental health in representative population samples from Germany, Russia, and the United States, in addition to samples from students in China and Germany. An increase in anxiety and depression was observed only in the sample from China, but the result was considered mild since a vegetarian diet would explain only 1 percent of the variance in cases of depression and anxiety. In addition, the motivations that led Chinese students to adopt a vegetarian diet differed from those of the other groups studied, being more related to cultural and economic factors [88]. A study with Chinese elderly people also found a positive association between adopting a plant-based diet and depression compared to a meat-based diet. However, the correlation was observed only in men [89].

A French cohort’s cross-section study carried out a separate analysis by types of vegetarian diets, and identified a positive association between depressive symptoms and a fish diet and an ovo-lacto-vegetarian diet. However, no association was found with a vegan diet, which contradicts the idea that a stricter diet (excluding more or all animal products) would lead to more severe symptoms of depression [90]. The authors claim that differences in motivation (between vegans and other vegetarians) may have contributed to this group’s lack of association. In addition, the same study found a positive association between depressive symptoms and the exclusion of items from the diet, both for foods of animal and vegetable origin. That is, the more items excluded (not types of food, but number of products excluded), the greater the symptoms. Such a result could indicate that the higher levels of depression found in vegetarians in several studies could reflect an increase in risk related to diet restriction, and not necessarily to vegetarianism itself [90].

Another point that needs to be considered is that studies on depression in vegetarians are predominantly transversal, and therefore do not enable the determination of a cause-and-effect relationship. A study that evaluated mental disorders and adopting a vegetarian diet in the previous 12 months (through interviews with a population sample in Germany) also found a positive association between the two variables. However, the time difference between the beginning of both suggests that mental disorders preceded the change in diet, thus refuting the hypothesis that vegetarianism might cause mental disorders [91].

A systematic review study carried out by Medawar et al. [92] points out that, despite several health benefits related to adopting a vegetarian diet, its effect on mental health has yet to be properly studied. It is possible that nutritional deficiencies, such as lower levels of vitamin B12, contribute to worsening the nervous system’s health. On the other hand, a diet that favors a more balanced intestinal microbiota, such as a vegetarian diet, positively contributes to the maintenance of neurological functions due to its importance in modulating the gut-brain axis [92]. In a meta-analysis study published in 2016, it has also been observed that the consumption of fruits and vegetables is inversely associated with the risk of depression [93]. Vegetarians consume more fruits and vegetables than omnivorous individuals [17], and also tend to have better health markers and lower risk of other chronic diseases [94]. In view of this, the conflicting results on the relationship between vegetarianism and depression may reflect a lack of standardization with regard to diet quality and adequate intake (or supplementation) of nutrients in some of the studies, as well as the possibility already raised of reverse causality.

#### 3.2.2. Influence of the Psychological Domain on the Adoption of a Vegetarian Diet

##### Positive Influence

The main reason individuals decide to adopt a vegetarian diet is because of ethical/moral reasons [7,9], which is related to compassion and empathy towards the animals. Since some people feel that eating animal products is wrong, abstaining from their consumption could contribute to a better psychological state. Adopting a vegetarian diet can bring about positive feelings, such as altruism and a sense of purpose, while the pursuit of such guilt-free peace of mind could also positively influence one’s choice to adopt a vegetarian diet. A study conducted by Antonetti and Maklan [95] showed that experiencing either guilt or pride could change consumers’ behavior and their intention to purchase more sustainable products. Building on that, feeling guilty about eating animal products could lead to a behavioral change, and feeling proud of doing it could reinforce the maintenance of a vegetarian diet.

Moreover, some individuals adopt a vegetarian diet due to spiritual or religious reasons [7]. Spirituality is a concept related to people’s quest for the meaning in life and a connection to a higher or sacred power. On the other hand, religiousness is related to the degree in which an individual believes, follows, and practices a religion, which might influence how one chooses to live their lives [96]. An individual who follows a religion that preaches abstention from animal products might feel encouraged to adopt a vegetarian diet. Good adherence to the diet could, in this case, be a positive psychological reinforcement, as it would be in line with their own beliefs. As it has already been demonstrated, high levels of spirituality and religiosity are associated with better social, psychological, and environmental QoL outcomes [96]. 

##### Negative Influence

Despite the positive outcomes related to the adoption of a vegetarian diet, some challenges can be found. For many, the barriers to adopting vegetarianism outweigh the possible benefits, and may prevent them from taking that step. Studies corroborate the evidence that attachment to the taste of meat constitutes an obstacle to adopting vegetarianism [97,98]. In addition, other barriers may be considered, such as the fear that a vegetarian diet could be nutritionally inadequate or monotonous, or that it may not favor satiety; the belief that preparing vegetarian meals is harder; difficulties in finding options when eating in restaurants; living with people who eat meat; and a lack of knowledge about meat-free eating [97,99,100,101]. Especially among men, meat is considered a “comfort food”, and its intake is associated with strength, muscle building, and masculinity. These beliefs represent a barrier to reducing meat consumption, as demonstrated by a study with soldiers from Norway who evaluated their perception of the implementation of the “Meatless Monday” program [97]. The program is a worldwide campaign, adopted in more than 40 countries, which aims to make people aware of the advantages of reducing meat consumption [102]. 

These results are in line with older studies conducted by Lea et al. [103,104]. Having a taste for meat was considered the main barrier for the adoption of a vegetarian diet, but other important factors have also been described, such as, for example, difficulties in changing one’s eating pattern; the fact that family and friends may still eat meat; little knowledge about the subject; and difficulties in finding vegetarian options when eating out [103]. 

Moreover, according to another study from Lea et al. [104], some of the factors that prevent or hinder the adoption of a plant-based dietary pattern are related to one’s family (family members or close people do not adopt this eating pattern); convenience (difficulty finding options or preparing food); health (fear of iron, protein and other nutrient deficiencies); cost and lack of options for eating out; and lack of information about vegetarianism. The low prevalence of adopting a plant-based diet among the participants demonstrates that several factors discourage its adoption—even though it is a more flexible dietary pattern than a vegetarian diet.

All these barriers interconnect with the social domain, as they are influenced by the social context in which an individual is inserted. Nevertheless, the negative psychological effects refer to how individuals react to these fears or barriers, which might negatively affect their choice of adopting a vegetarian diet. As described by Schmitt et al. [105], the perception of discrimination, both about an individual and a group, has an impact on well-being, with potential psychological consequences (contributing to mental stress, anxiety, depression) and affecting other aspects, such as self-esteem, humor, and satisfaction with life [105].

### 3.3. Social Domain 

The social domain related to QoL includes personal relationships and social support [28]. In fact, having good social connections is essential for mental health and well-being, positively influencing one’s QoL. In this case, the consequences of adopting a vegetarian diet have to be analyzed based on the social and cultural group in which an individual is inserted, as well as the attitudes of close people towards vegetarianism.

#### 3.3.1. Influence of Adopting a Vegetarian Diet on the Social Domain

##### Positive Influence

Unlike other dietary patterns, vegetarianism goes beyond the definition of one’s food choices. Rather, it is defined as a social identity, which consists of how a person identifies themselves in terms of the social group in which they believe to belong. A study conducted with young vegan women revealed that not only did they identify with the diet, but they also passionately engaged in a “vegan lifestyle”. The choice of becoming a vegan had positive effects in many different ways, including social relationships, and identification and sense of connection with the vegan subculture [106]. Therefore, the choice of following a vegetarian diet can enhance one’s connection with other people who share the same life philosophy [107], strengthening social bonds and positively influencing one’s QoL (Figure 1).

##### Negative Influence

Many of those who decide to adopt vegetarianism suffer rejection from others and are victims of stereotyping and discrimination. Such negative attitudes towards vegetarians and vegans are known as “vegaphobia” or “veganophobia”, a term already spread in the scientific literature. A possible explanation for the discrimination against vegetarians and vegans is related to the cognitive dissonance suffered by individuals who eat meat. In this context, cognitive dissonance refers to the contradiction experienced by individuals who like animals and feel compassion for them, but, at the same time, consume meat. Therefore, individuals who eat meat may discriminate against vegetarians not out of fear or dislike, but because they represent an affirmation that eating meat is not necessary and is, therefore, unjustified [108].

In order to avoid conflict and embarrassment, many vegetarians prefer to omit their dietary choice. In fact, social aspects are so relevant that the greatest reason why vegetarians make exceptions and eat meat is due to pressure from friends, family, and coworkers. According to Rosenfeld and Tomiyama [109], in a qualitative study that evaluated dieters’ motivations to break their diet, 51 percent of individuals reported having already eaten meat after adopting vegetarianism. In general, their justifications do not involve missing meat itself, but rather an attempt to avoid uncomfortable situations in a social context. The fear of being rude or offending some family culture or tradition, the need to make a good impression, or the fear of being stigmatized are some of the most important factors that lead vegetarians to stop following their diets momentarily. Such a study reinforces the idea that vegetarianism goes far beyond a dietary choice, creating a social identity that influences the entire context in which an individual is inserted [109].

The negative consequences of a vegetarian identity usually have a stronger impact on vegans than vegetarians because the former suffer more rejection and are viewed more negatively by omnivores [23]. Such discrimination comes not only from nonvegetarian people, but also from the media, as demonstrated by Cole and Morgan [110] in a study that evaluated how veganism was reported in UK newspapers. Such a study concluded that the media tends to present vegans as sentimentalists, fanatics and extremists, in addition to mocking veganism and considering it impossible to maintain in practice.

#### 3.3.2. Influence of the Social Domain on the Adoption of a Vegetarian Diet

##### Positive Influence

Vegetarians and vegans also showed more adherence to their diet when compared to individuals who follow a paleo, gluten-free, or weight-loss diet. Social identification was an important predictor of adherence in both quantitative and qualitative analyses. According to Cruwys et al. [83], vegetarians and vegans described their diet not as an individual choice, but as a manifestation of their social ethics. Ethical and moral concerns were considered the most important facilitators of diet adherence, and a lack of adherence would go against the group’s moral code. Feeling part of a social group can also positively influence how strictly one sticks to a dietary pattern. The sense of belonging and the in-group social reinforcement could make it easier for individuals to maintain their dietary patterns, provided they feel supported by the group.

Vegetarians that have a close circle of vegetarian contacts (friends, family or coworkers) have been shown to have higher QoL than those who do not [13]. In this case, they can be positively influenced by their social environment. Moreover, just as the social context in which vegetarians are inserted may influence their adherence to the diet, individuals who eat meat may also be influenced by living with vegetarians. In their study, Geerts, Backer, and Erreygers [108] described some characteristics of meat-consuming individuals, with emphasis on the fact that meat consumption is considerably lower among those living with vegetarians in the same household. In addition, discrimination against vegetarians was less common among individuals who had vegetarians in their household or circle of friends. Thus, greater acceptance and lower levels of veganophobia among meat consumers (resulting from their close contacts with vegetarians) may have a positive influence on other individuals’ feeling more comfortable when adopting a vegetarian diet.

##### Negative Influence

Cultural aspects are relevant predictors of meat consumption. The consumption of different species of animals varies between cultures. Animals considered suitable for consumption in some countries may not be seen in the same way by individuals of other nationalities. As demonstrated by Ruby [111], in countries considered individualistic (such as the United States and Canada), a feeling of disgust is the primary attitude of certain individuals when faced with the idea of eating certain animals. On the other hand, in more collectivist nations, such as China and India, cultural norms influence individual emotions and the sense of morality, being the greatest predictor for not consuming meat.

Moreover, gender differences may also influence one’s choice of eating or avoiding animal products. Meat consumption is usually seen as a symbol of masculinity and dominance over other species in several cultures where meat is considered a proper food for men [23,97]. In addition, men tend to eat less fruits and vegetables; care less about the nutritional properties of the food they eat; and agree more with the belief that a healthy diet needs to include meat [7,112]. According to Rosenfeld and Tomiyama [98], men are more resistant to adopting a vegetarian diet, mainly because they believe that a meatless diet would not be tasty. In addition, women are more likely to believe that meat consumption is harmful to the environment and that adopting vegetarianism is a plausible and healthy choice [113]. In fact, large population studies such as the Epic-Oxford [114] and the Adventist Health Study 2 [115] identified a higher proportion of females among vegetarians, with 78 percent and 65 percent of the sample consisting of women. 

Such gender differences may influence the adoption of vegetarianism depending on the sociocultural context in which an individual is inserted. A study by Ruby et al. [116] with participants from Argentina, Brazil, the United States, and France (countries that are among the largest consumers of beef in the world) revealed that men consume beef more frequently and enjoy the taste of it more, while women show more negative attitudes towards the consumption of red meat, such as disgust. The same study also demonstrated that there are cultural differences related to the acceptance of vegetarianism. American women showed greater admiration for vegetarianism, while French women were the ones who admired vegetarians the least. Participants from Brazil and Argentina, considering the entire sample, demonstrated more positive attitudes toward beef consumption, followed by participants from France and, finally, from the United States [116].

### 3.4. Environmental Domain 

The environment in which an individual is inserted also exerts an important influence on their QoL. Living in a safe and healthy environment, with proper social care and an efficient transport system, opportunities for acquiring new information and skills, as well as recreation/leisure areas, are all relevant factors. Moreover, having good financial resources can positively contribute to a good QoL. On the other hand, factors that have a negative impact on the environment, such as pollution and climate change, could also negatively affect one’s QoL [28]. 

#### 3.4.1. Influence of Adopting a Vegetarian Diet on the Environmental Domain

##### Positive Influence

Following a more sustainable diet, which will contribute to a healthier environment, could positively influence QoL (Figure 1). In general, plant-based diets are more sustainable than those based on animal foods, as they require fewer natural resources for food production and have a lower impact on the environment. An omnivorous diet is estimated to require 2.9 times more water, 2.5 times more energy, 13 times more fertilizers, and 1.4 times more pesticides than a vegetarian diet [117]. In addition, meat and dairy production contribute 80 percent of all gas emissions from food production, and 24 percent of total greenhouse gases coming from food. Livestock production uses about 70 percent of all agricultural land globally, and consumes 29 percent of all water spent on agriculture [118].

Regarding the analysis of different types of diets, the data from 34 articles gathered in a systematic review showed that the more a diet is plant-based, the more sustainable it is. The vegan diet was considered the most sustainable of all, with the lowest greenhouse gas emissions and the least environmental impact, especially when based on locally produced foods and with a lower consumption of ultraprocessed meat substitutes. Ovolactovegetarian diets have a greater environmental impact than vegan diets, and it has been shown that 40 percent of greenhouse gases from ovolactovegetarian diets are attributed to the consumption of dairy products [118].

The production of animal-origin food is very inefficient in terms of energy, as it requires the use of many resources (water, energy, land, food) to keep animals alive. The animals themselves use much of the energy and nutrients in the form of food to maintain their metabolism, whereas only a small part of it is actually stored and converted into food for humans in the form of meat. This amount of energy wasted during production, standardized through the rate of the conversion of energy into protein, varies considerably from one animal to another. Whereas 4 calories from fossil fuels are required for each calorie of chicken protein that is produced, 40 calories are required for the production of 1 calorie of beef protein. For pork and dairy production, the rate is 14 fuel calories for each calorie of protein. In the case of eggs, the value is similar to that of beef (39 calories). On average, the energy used to produce each gram of animal protein (25 kcal/g) is 11 times greater than that used to produce vegetable proteins (2.2 kcal/g) [119].

In general, in the case of plant-origin foods, the higher the protein concentration, the greater the energy efficiency (which means that such foods need less energy to provide greater amounts of protein, as they are more concentrated in protein). Such an association does not exist for foods of animal origin, as their energy demand is very high—in fact, a decline in energy efficiency is observed as protein concentration increases (that is, foods with a higher protein concentration are those that demand more energy) [120,121].

According to Aleksandrowicz et al. [122], the change from a typical Western diet to more sustainable food patterns could reduce greenhouse gas emissions and land use related to food production by up to 80 percent, in addition to a 50 percent reduction in water use. In that study, all diets involved reducing or replacing animal foods with others of plant origin (such as, for example, vegetarian, vegan, Mediterranean and pescatarian diets), in addition to replacing the consumption of ruminant animals with monogastric animals [122]. Similar results were observed in a study by Rosi et al. [12] in Italy, which showed that vegetarian diets (ovolactovegetarian and vegan) had a lower ecological footprint in the three aspects assessed: CO_2_ production, water consumption, and land use. Corroborating these data, a global analysis of different dietary strategies to reduce the environmental impact and improve health estimated that, in developed countries, the replacement of animal foods with plant-origin foods could reduce the number of premature deaths by up to 12 percent, and greenhouse gas emissions by up to 84 percent [123].

#### 3.4.2. Influence of the Environmental Domain on the Adoption of a Vegetarian Diet

##### Positive Influence

Environmental issues are part of the motivations that lead individuals to reduce meat consumption or adopt a vegetarian diet. The concept of sustainability applied to food refers to a diet that, in addition to being nutritionally adequate and healthy, respects biodiversity and ecosystems, is accessible, culturally accepted, and contributes to preserving natural resources [124]. 

A motivation to live in a healthier and more sustainable environment may positively influence people to adopt and maintain a vegetarian diet, as it has already been proved that a more plant-based diet has a lower environmental impact when compared to animal-based diets [122]. Individuals who are naturally engaged in sustainability and environmental issues are more likely to have positive feelings related to a sense of altruism achieved from adopting a vegetarian diet. The possibility of protecting their own environment and contributing to a better world can bring a sense of purpose in life [125], which could positively influence diet adherence and QoL.

##### Negative Influence

Adopting a vegetarian diet may depend on other factors beyond an individual’s will. Economic aspects, both at the global level (economic situation of the country) and the individual level (income and social status), could influence food choices. In general, the lower the income, the greater its influence on food. People with higher income suffer less from fluctuations in food prices and are more demanding in their choices. Likewise, in poorer countries, the consumption of certain foods is highly influenced by their prices, which does not occur with the same intensity in developed countries [126]. The influence of economic aspects on the nutritional quality of a diet is quite variable. For example, it has been shown that increased income leads to a higher intake of fruit. However, the same increase might lead to eating out more often, or consuming more processed foods, in addition to eating more meat and fewer legumes [126]. Moreover, a cross-sectional study carried out in the United States showed that lower income levels were associated with poorer quality of food—in particular, lower consumption of fruits and vegetables and higher consumption of sugary drinks and frozen desserts [127].

The economic context is one of the factors that may influence the adoption of vegetarianism. On the one hand, the price of animal-origin foods may cause individuals to reduce their consumption. A study carried out in Canada found that an increase in meat price led 37.9 percent of individuals to reduce or eliminate their consumption. Still, as it is a food that is part of local culture, individuals value meat consumption more than any other food group. Therefore, despite economic issues, cultural aspects may also be considered an important barrier to reducing meat consumption [128]. In Australia, it has been shown that price increases are the biggest motivators for reductions in meat consumption, a factor that was considered more relevant than health, religious, ethical, and environmental aspects, among others [129]. Therefore, understanding the economic context in which individuals live is essential for understanding the motivations that lead them to reduce their meat consumption and possibly adopt vegetarianism.

Reducing meat consumption also depends on access to various plant-origin foods, which is also limited by economic issues. In Brazil, for example, the consumption of fruits and vegetables is influenced by prices and family income, with the cost burden being indicated as the primary barrier [130]. Data from the Brazilian Household Budget Survey (POF) showed that individuals from lower income groups spend a higher percentage of their budget on food. Families with a monthly income of up to BRL 1908.00 spend 22.6 percent of their household budget on food, compared with only 7.6 percent among families whose monthly income exceeds BRL 23,850.00 [131]. One of the barriers to adopting a vegetarian diet is the perception that it would be more expensive [98]. However, a vegetarian diet could be considered cheaper than an omnivorous diet, since meat is often the most expensive food item. In Brazil, a national survey from 2017–18 revealed that over 20 percent of all household food expenses were spent on “meats, viscera and fish”, a percentage higher than to any other food item [131]. Still, a vegetarian diet could become more expensive when more meat-substitute foods (which are less accessible) are consumed [132].

Another factor that could hinder the adoption of a healthy vegetarian diet is the logistics involving access to fresh fruits and vegetables. As they are perishable foods and are usually eaten fresh (unlike meats and other foods, which are often frozen and stored for longer), many types of fruits and vegetables require more frequent trips to the market, and adequate storage to minimize losses. Therefore, the consumption of fresh fruits and vegetables could be affected by people’s lack of time to purchase these foods frequently, and by losses resulting from inadequate storage. In other words, the perishability of fruits and vegetables could generate a cost increase. In addition, especially among low-income individuals, a more restricted access to fresh food is a factor that negatively influences its consumption [133]. Moreover, lower education levels could also negatively influence one’s decision to adopt a vegetarian diet, as a positive association has been demonstrated between higher educational levels and the adoption of a vegetarian diet [114,134]. In view of this, educating individuals to make healthier and more economically viable choices could encourage more people to adopt vegetarianism. Public policies that help reduce prices and facilitate access to fruits, vegetables, and other plant-origin foods could also help more people to reduce their meat consumption.

## 4. Vegetarians’ Quality of Life

A vegetarian diet’s effect on QoL was assessed in a cross-sectional study carried out with runners. A convenience sample was selected from German-speaking countries, namely Germany, Switzerland and Austria, and a total of 281 individuals (158 vegetarians and 123 omnivores) participated in the study. The instrument used to assess QoL was the WHOQOL-BREF, which was applied virtually to the study subjects. The results showed that all participants scored high on QoL, regardless of the type of diet adopted, with no difference between groups. Therefore, it was concluded that runners have high levels of QoL, and that a vegetarian diet was as good as an omnivorous diet for this population segment [135].

In Brazil, a specific questionnaire to evaluate the QoL of vegetarians was developed and validated, since other studies used only general questionnaires or others that were not specific to vegetarians [13]. The responses showed that vegetarians have satisfactory levels of QoL (average scores between 70 and 80 on a 100-point scale). Among the different types of vegetarians, vegans were the ones with the highest scores. Other factors that had an influence on participants’ QoL included their age, how long they had been following a vegetarian diet, and whether they had other vegetarians in their close circle of contacts [13].

In a clinical trial conducted with diabetic patients, the effect of a vegetarian diet on their QoL and eating behavior was compared to a standard diet used to treat type 2 diabetes. QoL was assessed using the Obesity and Weight-Loss QoL questionnaire (OWQOL) and Weight-Related Symptom Measure questionnaire (WRSM). Both diets led to positive effects on QoL and mood, but the effect was stronger in the group that followed a vegetarian diet, demonstrating that such a dietary pattern can have positive effects not only on the physical health, but also on the mental health of patients with type 2 diabetes [136].

Older studies [137,138,139] show similar results, with positive QoL outcomes when individuals were exposed to a vegetarian diet. Katcher, Ferdowsian, Hoover, Cohen, and Barnard [137] developed a workplace study in a US-based company as part of a health promotion program, in which volunteers adopted a vegan diet for 22 weeks. At the beginning and the end of the period, individuals answered the Food Acceptability Questionnaire—FAQ (SF) and the Work Productivity and Activity Impairment questionnaire (WPAI). The responses to the questionnaires showed that individuals who adopted the vegan diet reported improvement in general health, physical fitness, mental health, vitality and overall satisfaction with the diet, in addition to the reduced cost of food items. However, they reported more difficulty in finding options when eating out. Still, the vegan diet was effective in improving the participants’ QoL. QoL was also assessed in a study conducted at a health institute in the United States that offers a raw vegan diet to visitors and guests. Participants who remained at the institute for at least a week and who would maintain the raw vegan diet after leaving the institute were selected. A QoL analysis was performed at the beginning of the study and 12 weeks after the intervention, with a questionnaire that evaluated individual satisfaction with taste, food cost, convenience (ease of buying, planning and preparing food), and self-care perception. Individuals who followed the raw vegan diet for 12 weeks were compared to those who did not. There was an improvement both in the parameters of general QoL (assessed by SF-36), as well as in the QoL associated with changes in the diet, cost aspects and the perception of self-care. This shows the positive effect that this type of food can have in QoL, when used as a clinical treatment [138]

A study conducted in the United States by Barnard, Scialli, Bertron, Hurlock, and Edmonds [139] assessed the acceptability of a low-fat vegan diet in women. The study was carried out with 35 nonmenopausal women divided into two groups: one adopting the diet for a period equivalent to two menstrual cycles, and the other group not following any diet, with a crossover design. The low-fat vegan diet had high adherence and good acceptability, although the participants reported that maintaining the diet required more effort. They also reported weight loss and improved sleep, digestion and energy levels, which can positively contribute to improving QoL.

## 5. Summary of Knowledge and Future Directions

Adopting a vegetarian diet can have a positive influence on all four QoL domains. Better health outcomes and lower rates of noncommunicable diseases have a positive impact on the physical domain. Positive feelings associated with doing something good, together with a feeling of belonging or stronger in-group bonds created with the vegetarian community, have a positive effect on the psychological and social domains, respectively. Finally, the lower environmental impact of vegetarian diets benefits the environmental domain.

On the other hand, negative effects on QoL might also result from adopting a vegetarian diet. Despite better overall health, a nonbalanced vegetarian diet could lead to nutritional deficiencies that would be detrimental to health, affecting the physical domain. As vegetarians are still a minority group, rejection and stigmatization from nonvegetarians may have a negative impact on the social domain. The psychological and mental effects of a vegetarian diet are not clear, although some studies point to an increased risk of depression.

Several aspects of different QoL domains can also have an impact on one’s decision whether or not to adopt a vegetarian diet. Improving one’s health can be an important motivator to try a vegetarian diet. Ethical/moral and religious/spiritual reasons are important psychological aspects that can lead to the adoption of vegetarianism, while an attempt to reduce one’s environmental impact can motivate someone to adopt such a diet. Becoming part of a social group and achieving a sense of belonging can also be a trigger for someone to become vegetarian.

Just as some individuals might feel motivated to follow a vegetarian diet for a number of different reasons, others might feel discouraged due to psychological, social, or environmental factors. A fear of being stigmatized or excluded from their social group could hinder one’s intention of becoming a vegetarian. Moreover, cultural aspects that enhance meat consumption could have the same effect, together with the connection that people make between meat and masculinity. Finally, since the adoption of an alternative dietary pattern also relies on environmental factors, such as food availability and economics, individuals may face difficulties when adopting a vegetarian diet if they lack a good supply of plant-based food options.

## 6. Conclusions

In conclusion, vegetarianism can either influence or be influenced by different QoL domains. The choice of adopting a vegetarian diet can have positive consequences, such as better physical health, positive feelings related to the adoption of a morally correct attitude, an increased sense of belonging (to a vegetarian community) and lower environmental impact. On the other hand, factors that go beyond an individual’s control, such as the environment and social/cultural group in which they are inserted, as well as gender-based differences, economic aspects, and limited access to a wide variety of plant-based foods, can negatively impact the QoL of those choosing to abstain from meats or other animal products. Despite the low number of studies on vegetarianism and quality of life, the existing evidence points toward a more positive impact. It is important to understand all the effects of adopting a vegetarian diet—beyond its nutritional aspects. Not only do studies in this area provide more consistent data, but they may also contribute to mitigating all factors that might prevent individuals from adopting a vegetarian diet, or that may have a negative impact on the quality of life of those who already follow it. Further studies are necessary to understand how strongly these connections between QoL domains and vegetarianism can influence the individuals who adopt this dietary pattern.

## Data Availability

The study did not report any data.
